# The relationship between transformational leadership and work engagement in governmental hospitals nurses: a survey study

**DOI:** 10.1186/2193-1801-3-25

**Published:** 2014-01-14

**Authors:** Davood Hayati, Morteza Charkhabi, AbdolZahra Naami

**Affiliations:** Department of Psychology, Shahid Chamran University, Ahvaz, Iran; Department of Psychology, University of Verona, Lungadige Porta Vittoria, Verona, 17 - 37129 Italy

**Keywords:** Transformational leadership, Work engagement, Hospital nurses

## Abstract

The aim of this study was to determine the effects of transformational leadership and its components on work engagement among hospital nurses. There are a few set of researches that have focused on the effects of transformational leadership on work engagement in nurses. A descriptive, correlational, cross-sectional design was used. In this study, 240 nurses have been chosen by stratified random sampling method which filled related self-reported scales include multifactor leadership questionnaire (MLQ) and work engagement scale. Data analysis has been exerted according to the statistical method of simple and multiple correlation coefficients. Findings indicated that the effect of this type of leadership on work engagement and its facets is positive and significant. In addition, the research illustrates that transformational leaders transfer their enthusiasm and high power to their subordinates by the way of modeling. This manner can increase the power as a component of work engagement in workers. Idealized influence among these leaders can result in forming a specific belief among employees toward those leaders and leaders can easily transmit their inspirational motivation to them. Consequently, it leads to make a positive vision by which, and by setting high standards, challenges the employees and establishes zeal along with optimism for attaining success in works. regarding to the results we will expand leadership and work engagement literature in hospital nurses. Also, we conclude with theoretical and practical implications and propose a clear horizon for future researches.

## Introduction

Modern day organizations are in a constant state of flux and often experience large-scale change; hence the guidance of visionary leadership is vital to the success of any business. According to Jones and Harter (2005), “engagement leads to human benefits for the individual who experiencing it,” (p. 79) and since supervisors are most likely to have daily contact and influence over the subordinate, they are also most important to the discussion of leadership because of their ability to influence employees to stay motivated and engaged at work (Koppula, 2008). Based on investigations, traditional methods of leadership and management in today’s wavy world do not work anymore (Leithwood, 1992; Liontos, 1992). So, behavioral science researchers are seeking for appropriate managerial ways to soar up the level of employee’s commitment and enthusiasm toward working. Nowadays organizations need such managers and leaders that can develop zeal and commitment among subordinates by using behavioral and personality characteristics such as charisma, the ability of high influence and extended vision which will lead to utilize the total amount of talent and effort behalf of achieving organizational goals. These leaders are called transformational leaders (Bass and Avolio, 1997). Transformational leaders can encourage employees toward gaining valuable organizational goals include higher productivity, presenting better services and solving social problems (Spector, 2004). They prepare real and challenging purposes and enlarge the sense of identification, competency and worthiness among jobholders.

### Transformational leadership and work engagement

The Bass et al. (2003) divides transformational leadership into four areas which embrace: Idealized influence, Inspirational motivation, Intellectual stimulation and Individualized consideration. Idealized influence; means making a glorious image along with profound and self-confidence based respect in presence of employees. Inspirational motivation; refers to leaders that draw a strict and positive view of future for their subordinates and stimulate them to go toward organizational aims and chief missions. Intellectual stimulation; in this manner, leader emphasizes on actualizing creativity and invention and using novel ways in doing works. Individualized consideration; this dimension represents the leader’s own attention to subordinates and treating them in the best route.

Another variable in this study is work engagement which is composed of three dimensions that include absorption, vigor and dedication. Absorption means concentration and being engrossed in people’s work, whereby passing time will be intangible and being detached from the job has some difficulties for them (Gonzalez-Roma et al., 2006; Langelaan et al., 2006; Liorens et al., 2007). Furthermore, it is pleasurable to have job experience for individuals. They do that, only for having that and paying high expenditure for job is not such important issue which it is for the others (Bakker and Demerouti 2007). Vigor is another aspect of work engagement that implies high levels of energy and mental resilience while working. There is also a determined investment in the actual work, together with high levels of persistence even when faced with difficulties (Schaufeli and Bakker, 2004). Salanova et al. (2005) propose that this aspect can be determined based on Atkinson’s motivational theory (Atkinson, 1965). Motivation is strength of doing work or resistance against that. So, strength and resistance are addressed as aspects of work engagement and their concept is constant with popular definition of motivation (Latham and Pinder, 2005; Steers et al., 2004). The third dimension is dedication that refers to a sense of significance, enthusiasm, inspiration, pride, and challenge (Schaufeli and Bakker, 2004, 2010). In another word, this aspect can be seen when a person has a great involvement with his or her job (Brown, 1996). Dedication has a lot of things in common with job involvement which is known as the amount of attachment and identification with job. Previous studies have shown that job resources (e.g., autonomy; for reviews, see Bakker, 2009; Halbesleben, 2010) and personal resources (e.g., self-efficacy; Xanthopoulou et al., 2007; Xanthopoulou et al., 2009), due to their motivational potential, are important antecedents of work engagement.

### Literature review

Several studies have examined the relationship between leadership and employee engagement, however, only a few have attempted to study the linkage specifically between the multidimensional constructs of transformational leadership and employee work engagement. The concept of work engagement has gained momentum because of its predictive value for job performance (Bakker, 2009; Schaufeli and Salanova, 2007). Leadership plays an important role while dealing with diverse mix of employees that are increasing rapidly in organizations (Sparks et al., 2001), and transformational leadership style help followers to coordinate with each other effectively increases followers’ satisfaction level (Shibru, 2011). Transformational leadership has positive impact on employees’ behaviors (Al-Swidi et al., 2012). This research study focuses on Bass’s conceptualization and measurement of leadership. Bass (1999) as one of the foremost researchers of leadership studies categorized leaders as being either transactional or transformational and suggested that transformational leaders displayed “superior leadership performance” (p. 21) when they appealed to the elevated spirit of individuals, to motivate them to transcend their individual self-interest for the greater good. Other definitions of transformational leadership have been proposed by Avolio et al. (1999) who defined transformational leaders as being charismatic and influential in their ability to make employees do more than what was expected of them at work. Likewise, Seltzer and Bass (1990) asserted that transformational leaders commanded by inspiring and encouraging their subordinates to use novel methods to solve problems. Several adjectives have also been used in workplace literature to describe transformational leaders, such as charismatic, powerful, influential, trustworthy, confident, inspirational, motivating, exciting, world-class, and considerate (Bass et al., 1987; Bass, 1985a).

In the latest investigations, the relationship between transformational leadership and organizational effectiveness (Moore, 2008), job satisfaction and organizational commitment (Pillai et al., 1999), turnover (Chan, 2005), withdrawal behaviors (Walumbwa, 2005), job performance (Bass et al., 2003), and job motivation (Macey and Schneider, 2008) had been resulted. Determining the relationship between transformational leadership and work engagement is required in research field. In their recent review, Macey and Schneider (2008) listed various different definitions of work engagement. Work engagement is the amount of energy a person spends for doing his or her own works, and also, the earned effectiveness and efficiency of that work (Maslach et al., 2001). However, and in line with Schaufeli and Bakker’s (2004) definition, work engagement may also be observed as a state that may fluctuate within the same person. In this case, Sonnentag et al. (2010) demonstrated that generally engaged employees may have off-days, since “not all days are created equally”. However, it is indicated that work engagement has relationship with high productivity, and also, meeting customers’ needs and pleas (Brown, 1996). It worth mentioning to name some antecedents of work engagement include job stress (Demerouti et al., 2001), social, mental and physical characteristics of job (Bakker et al., 2005), education and independence at work (Bakker et al., 2007), and work-family conflict (Greenhaus and Beutell, 1985). So, the main purpose of this study is to investigate the interrelationships between the multidimensional constructs of transformational leadership, as conceptualized by Avolio et al. (1999), and employee work engagement, as conceptualized by Schaufeli et al. (2002).

## Materials and methods

### Participants and procedure

In this study participants were employed from five public hospitals in Khuzestan province of Iran. Among all the nurses, 240 ones (185 women and 55 men, M_age_ = 25) were selected by stratified random sampling method and they were asked to fill the multifactor leadership questionnaire (MLQ) and work engagement scale. All questionnaires were delivered to participants by researchers. On the front page there was an information letter, in which the study was introduced and confidentiality of the responses was emphasized.

### Hypotheses

Current research was carried out to evaluate one main hypothesis and several subsidiary hypotheses which are presented in the below:

1. Transformational leadership is associated positively with work engagement.
2. Transformational leadership is associated positively with vigor.
3. Transformational leadership is associated positively with dedication.
4. Transformational leadership is associated positively with absorption.
5. Transformational leadership components are predictors of vigor.
6. Transformational leadership components are predictors of dedication.
7. Transformational leadership components are predictors of absorption.

### Tools

#### Multifactor leadership questionnaire (MLQ)

Transformational leadership was measured by multifactor leadership questionnaire designed by Bass and Avolio (1997) which evaluates two leadership styles include transactional and transformational leadership; but in this study, we just used the transformational one. This contains 20 questions that the portion of inspirational motivation, intellectual stimulation and individual consideration are equal. It means that every one of last components would be assessed by 4 questions, but, it measures idealized influence by 8 questions. Bass and Avolio (1997) reported its reliability based on 14 studies in financial, industrial, military and medicine occupations between 0.81 and 0.94. The validity is measured by correlating this scale to the leader behavior description questionnaire (LBDQ). The Leader Behavior Description Questionnaire (LBDQ) was developed by the staff of the Personnel Research Board in the Ohio State Leadership Studies, directed by Dr. Carroll L. Shartle and all the validity results were significant and satisfactory.

#### Work engagement scale

Schaufeli et al. (2002) developed A self-report questionnaire, consists of 17 items, which measure the three underlying dimensions of work engagement: vigor (six items), dedication (five items), and absorption (six items). At first it consisted of 24 items, but after psychometric testing, seven unsound items were removed and 17 items were retained. A 6-pint Likert scale was used with answers ranging from 1 (never) to 7 (always). Overall reliability (Cronbach alpha) was 0.73. Internal correlation between aspects of questionnaire (vigor, dedication and absorption, respectively) was reported 0.78, 0.91 and 0.73. Correlating this questionnaire with Maslach Burnout Inventory (MBI), Schaufeli and Salanova estimated its validity and find it out - 0.38. In addition, all the subscales of burnout had negative significant relationship with work engagement components.

### Statistical analysis

Data analysis was carried out by the methods of descriptive statistics include mean and standard deviation, along with inferential statistics methods include simple correlation to evaluate the correlations between all studied variables and multiple regression to assess the relationships between components of transformational leadership, as independent variable and those of work engagement, as dependent one. It is imperative to note that errors of accepting or rejecting the null hypothesis were considered in this research. Research tends to accept that when p ≤ 0.05, then acceptable levels of significance have been achieved. Care should be taken not to make Type I (risk of false HO rejection) or Type II (risk of false *H*O acceptance) errors. To reduce the risk of these errors, the size of the study should be considered when determining significance (Rosenthal and Rosnow, 2008).

## Results

### Descriptive statistics and simple correlations

Table 1 presents mean scores, standard deviations and simple correlations among the studied variables. Demographic variables were non-significantly related with the studied variables and thus, were excluded from further analyses.

**Table 1:** **Means, standard deviations and simple correlations among study variables (N = 240 nurses)**

		M	SD	2	3	4	5	6	7	8	9
**1**	Transformational leadership	47.98	14.05	.57^**^	.87^**^	.73^**^	.91^**^	.70^**^	.59^**^	.57^**^	.40^**^
**2**	Intellectual stimulation	9.74	3.22	-	.29^**^	.13^**^	.30^**^	.58^**^	.41^**^	.54^**^	.35^**^
**3**	Inspirational motivation	10	3.62	-	-	.59^**^	.85^**^	.66^**^	.56^**^	.48^**^	.42^**^
**4**	Individual consideration	8.88	3.24	-	-	-	.64^**^	.41^**^	.35^**^	.42^**^	.16^**^
**5**	Idealized influence	19.19	5.97	-	-	-	-	.56^**^	.51^**^	.38^**^	.36^**^
**6**	Work engagement	57.73	17.99	-	-	-	-	-	.67^**^	.79^**^	.78^**^
**7**	Vigor	20.85	7.04	-	-	-	-	-	-	.30^**^	.24^**^
**8**	Dedication	16.59	5.92	-	-	-	-	-	-	-	.49^**^
**9**	Absorption	20.30	7.31	-	-	-	-	-	-	-	-

** p < .01.

As it can be observed, the highest mean and standard deviation values among all studied variables belong to transformational leadership (M = 47.98) and work engagement (SD = 17.99), respectively. As it is shown in this table, and based on hypotheses 1, 2, 3 and 4, which propose that transformational leadership has positive relationship with work engagement (r = 0.70), vigor (r = 0.59), dedication (r = 0.57) and absorption (r = 0.40), respectively, all aforementioned relationships are confirmed in p < 0.01 significance level. So, it is concluded that the hypotheses 1, 2, 3, and 4 are confirmed.

*Testing Hypothesis 5*: this hypothesis states that transformational leadership components have positive relationship with vigor. As it is shown in Table 2, results supported all the aforementioned relationships.

**Table 2:** **Results of multiple regressions related to components of transformational leadership and vigor**

Leadership component	Multiple regression	R square	β	t	P
Idealized influence	.504	. 254	.504	7.82	.0001
Inspirational motivation	.597	.356	.585	5.33	.0001
Intellectual stimulation	.641	.411	.248	4.07	.0001
Individual consideration	.642	.412	.022	.28	.77

P ≤ 0.05.

This table displays that multiple regression related to four components of transformational leadership and vigor is 0.64 which is significant (F = 30.95 & P < 0.001). Four components of transformational leadership explain about % 46 of vigor. Additionally, the results of β indicate that inspirational motivation has the biggest contribution in explaining the variance of vigor. These findings confirm hypothesis 5. *Testing Hypothesis 6*: this hypothesis anticipates the positive significant relationship between dedication and dimensions of transformational leadership. Mentioned results in Table 3 suggest that coefficient relationships between these variables vary from 0.25 to 0.59 (P < 0.001). In addition, data reported in Table 3 illustrate that multiple regression between transformational leadership dimensions and dedication is significant (MR = 0.69 & P < 0.001). These dimensions predict % 47 of dependent variable (dedication) whereby intellectual stimulation has the most contribution (β = 0.46). Then, this hypothesis is confirmed.

**Table 3:** **Results of multiple regressions related to components of transformational leadership and dedication**

Leadership component	Multiple regression	R square	β	t	P
Idealized influence	.379	.144	.379	5.50	.0001
Inspirational motivation	.492	.242	.572	4.81	.0001
Intellectual stimulation	.642	.412	.436	7.17	.0001
Individual consideration	.692	.479	.341	4.78	.0001

P ≤ 0.05.

*Testing Hypothesis 7*: this hypothesis predicts positive significant relationships between transformational leadership components and absorption. Presented results in Table 4 indicate that there are significant multiple correlations between four components of transformational leadership and absorption which inspirational motivation has the biggest amount of beta in explaining absorption’s variance. Namely, Table 4 displays that multiple regression between transformational leadership dimensions and absorption is significant (MR = 0.47 & P < 0.001). These dimensions predict % 22 of dependent variable (absorption) and inspirational motivation has the most contribution (β = 0.44).

**Table 4:** **Results of multiple regressions related to components of transformational leadership and absorption**

Leadership component	Multiple regression	R square	β	t	P
Idealized influence	.337	.113	.337	4.79	.0001
Inspirational motivation	.417	.174	.448	3.60	.0001
Intellectual stimulation	.477	.227	.246	3.52	.001
Individual consideration	.486	.237	- .126	-1.45	.14

P ≤ 0.05.

## Discussion

The integration of the two research variables provided some insights into the possible relationships between the effects of transformational leadership and work engagement. The data collected in this study suggested that there is a correlation between the independent and the dependent variable. The results of this study suggest that dimensions of transformational leadership have positive significant relationships with diverse components of work engagement. In addition, the results of multiple regressions showed that considerable variance of work engagement dimensions is explained by transformational leadership components, especially inspirational motivation. The main purpose of this research study was to examine the relationship between transformational leadership and employee work engagement. Bernard Bass’s conceptualizations of leadership and the measures developed to quantify leadership were based on the premise that leadership could either be transactional or transformational. However, for the purposes of this study, only transformational leadership was investigated, and it was suggested that transformational leadership would likely be the most predictive characteristic of an optimal leader. Avolio et al. (1999) noted that transformational leaders embodied characteristics of being charismatic and influential in their ability to make employees do more than what was expected of them at work. Similarly, Bass (1985b) suggested that employees were more likely to devote additional extra effort at work, if they reported to a transformational leader who guided their employees by stimulating them and inspiring their trust. These findings are in long with previous studied researches about relationship between transformational leadership and other organizational variables such as Moore (2008), Chan (2005), and Pillai et al. (1999).

Also, May et al. (2004) propose that work engagement augments through psychological safety. Psychological safety is defined as a feeling of self-expression without the feeling of scaring of negative outcomes. They have suggested that directive and supportive leadership can soar up psychological safety. Not using aggressive and criticized judgment, transformational leaders determine performance standards and criteria for employees. Also, individual consideration can shove leaders to consider employee’s needs, pleas and aspirations. In this case, above-mentioned leaders can provide suitable place for ensuring psychological safety and it can lead to free-expressed view points and suggestions by workers. Naturally, in this situation, desirable climate of contribution would be provided, by which employee’s commitment, engagement and involvement will be augmented (Harter et al., 2003).

By increasing employees’ control and dependency and encouraging them to experience new issues and overcome on them, transformational leaders can also raise the amount of work engagement. Organizational researchers address the control as amount of domination on workplace from the viewpoint of time and the way of doing work (Lee and Brand, 2005). Theoretical infrastructure of this issue stems at job characteristics model (Hackman and Oldham, 1976). Based on this model, five job characteristics include skill variety, task identity, task significance, feedback and authority have play a critical role in growing work motivation. In this part, job authority has specific role. Evidence shows that job control has positive impact on job outcomes such as job satisfaction, performance, mental health and wok motivation (Lee and Brand, 2005). Additionally, transformational leaders can encourage the employees to creative thinking and tendency of being successful by which they can increase work engagement by building required energy and power among them (Terry et al., 2000). Intellectual stimulation of these leaders can permit the workers to pose the old assumptions, values and beliefs, and then, consider exploring new ways of doing works and also, proposing the ideas. Transformational leaders stimulate the sense of self-value, self-motivation, eligibility, internal motivation and achievement and success among their followers (Shamir et al., 1993). All these issues can make jobs enrichment and challenging and consequently, enhance job motivation.

## Conclusion

Research illustrates that transformational leaders transfer their enthusiasm and high power to their subordinates by the way of modeling (Brief and Weiss, 2002). This manner can increase the power as a component of work engagement in workers. Idealized influence among these leaders can result in forming a specific belief among employees toward those leaders. Thus, and as a result, the followers identify with the leaders and match themselves with leaders’ expectations and aspirations. So, leaders can easily transmit their inspirational motivation to them. Consequently, it leads to make a positive vision by which, and by setting high standards, challenges the employees and establishes zeal along with optimism for attaining success in works.

Previous research showed that resources, like quality coaching (Xanthopoulou et al., 2007), contribute to the work engagement of employees. To the best of our knowledge, the present study is one of the first to investigate whether the transformational leadership style influences the level of work engagement of the employee through the enhancement of personal resources. In a similar vein, a recent study by Zhu et al. (2009) has also focused on the relationship between transformational leadership and employees’ work engagement. These researchers proposed and showed that transformational leadership is related to follower work engagement, particularly when the follower is creative, innovative and proactive. The added value of the present study is that it helps to unfold the psychological mechanisms that underlie the transformational leadership-work engagement relationship, rather than the factors that determine the magnitude of this relationship.

Finally, the current study contributes to the leadership literature. Most research on leadership has focused on the organizational outcomes of a specific leadership style, such as performance and efficiency (Harter et al., 2002; Howell and Avolio, 1993). The present study demonstrates that daily fluctuations in transformational leadership may also influence employees’ work experiences (i.e., work engagement). Employees become more engaged to their work, when their supervisor is able to boost their optimism through his/her transformational leadership style. These results imply that personal resources and work engagement may be important in explaining the transformational leadership–performance link, considering the strong positive link between work engagement and performance (Xanthopoulou et al., 2009; 2008). Future studies should provide evidence for this latter process. Additionally, it should be mentioned that the effect improving the abilities of transformational leadership among leaders, not only increases work engagement of workers, but also leads to increment in their performance and it is a privilege for the organization as an outcome.

So, at the end, it can be noted that future research should concentrate on aspects of these two variables, namely, transformational leadership and work engagement; because considering variables in detail can have effective results rather than general consideration. Investigating different roles of such variables as personality can be a significant way to find out substantial results for improving organizational outcomes. Variables such as personality style and sex are recommended to be assessed as moderators for this relationship.

### Relevance to clinical practice

However, we can offer several suggestions for organizations to become more effective in leadership. Leaders and managers should be assisted to develop what is already known about leadership and link to this the ideal qualities that developing effective and active leadership behavior. Industrial and organizational psychologists who are involved in developing hospitals leaders and manager should comprehend that leaders and manager have different backgrounds, experiences and professional exposure, are at different stages of personal development and display clear preferences in terms of leadership styles. In addition, hospital leaders have different capabilities, tolerances, desires and motives. Recognizing these extensive differences provides a strong base for the development of leaders. The formal training of hospital leaders could also be reinforced by the implementation of a mentorship program whereby the leader is provided with constant developmental feedback on behavior and reactions. It is necessary for leaders to know the process of feedback and prepare them for the feedback they will receive. Regarding to this point that the customers of hospital nurses are patients, so nursing is viewed as one of the sensitive jobs and this position needs to motivated and engaged nurses. Transformational leadership can be used as a motivator factor and intervention for improving the work engagement of hospital nurses.
